# New research models and novel signal analysis in studies on preterm labor: a key to progress?

**DOI:** 10.1186/1471-2393-7-S1-S6

**Published:** 2007-06-01

**Authors:** Piotr Pierzynski, Edward Oczeretko, Piotr Laudanski, Tadeusz Laudanski

**Affiliations:** 1 Department of Pathophysiology of Pregnancy, Medical University of Bialystok, M.C. Sklodowskiej 24a, 15-276 Bialystok, Poland; 2 Faculty of Nursing, the Academy of Agribusiness in Lomza, ul. Wojska Polskiego 161, 18-402 Lomża, Poland

## Abstract

Preterm labor affects up to 20% of pregnancies, is considered a main cause of associated neonatal morbidity and mortality and is responsible for neonatal care costs of multimillion euros. In spite of that, the commercial market for this clinical indication is rather limited, which may be also related to high liability. Consequently, with only a few exceptions, preterm labor is not in the orbit of great interest of the pharmaceutical industry. Coordinated effort of research community may bring the change and help required to reduce the influence of this multifactorial syndrome on society. Between the novel techniques that are being explored in a SAFE (The Special Non-Invasive Advances in Fetal and Neonatal Evaluation Network) group, there are new research models of preterm labor as well as novel methodology of analysis of biological signals. In this article, we briefly describe new clinical and nonclinical human models of preterm labor as well as summarize some novel methods of data processing and analysis that may be used in the context of preterm labor.

## Background

Preterm labor now constitutes the major cause for neonatal mortality in developed countries. In spite of decades of ongoing research, its pathophysiology still remains not fully discovered [1,2], and consequently – the specific pharmacologic armory to treat preterm labour is not large. Notwithstanding multimillion euro costs of neonatal care, the annual percentage of cases of preterm birth is relatively limited. As a result, most of the pharmaceutical industry considers this segment of market as small one and which additionally is connected to high liability, meaning: not attractive [3]. However, this hides the fact that the *numbers *of preterm births in Europe reaches hundreds of thousands.

Ethical issues in clinical studies significantly limit the possibility of using placebo-controlled protocols. On the other hand, the application of alternative protocols drastically reduces the chances of drug candidates in the process of registration. The effect of combination of above-mentioned factors creates the situation where very few medicines are labeled for use in pregnancy, and especially, preterm labor. Considering the whole situation surrounding preterm labor, it appears that only a coordinated effort of research networks, such as SAFE (The Special Non-Invasive Advances in Fetal and Neonatal Evaluation Network) and those involved in preterm labour, is capable of bringing the new data that could be of potential use in discerning its pathophysiology and point to new pharmacological targets.

Therefore, in this setting there is much to be done towards solving this important problem. Here, we put forward new clinical and non-clinical models of uterine contractile activity together with new methods towards the detection and processing of biological signals that can be used as significant examples to build upon between collaborating partners.

## New research models of preterm labor

One of the major concerns in studies on preterm labor, especially on uterine contractile activity is that practically none of the models is adequately sufficient and easy to use – mice uteri lack V_1a _vasopressin receptor [4], rats present with spontaneous uterine contractility [5] and labor in ginea pigs is characterized by the desensitization of the progesterone receptor [6]. Studies on primates may be considered the most valuable [7], however, the accessibility of this model is significantly reduced by its costs and ethical issues. Consequently, there is a need for both *in vivo *and *in vitro *models that could closely reflect the reactivity of human myometrium/uterus, serving in pathophysiologic/pharmacologic studies.

Valuable information on the biology and pharmacology of uterine contractile activity may be provided by *in vitro *studies on uterine strips isolated from human pregnant/non-pregnant women as well as *in vivo *human or animal models [8-11]. One of the modifications of the *in vitro *model of contractility of human myometrium presented in the literature [12,13], may involve utilization of pregnant strips sampled from patients delivered preterm. In the study conducted by Pierzynski *et al. *[14] we confirmed that barusiban, a novel oxytocin antagonist effectively reduced oxytocin-induced contractions of both preterm and term myometrium. Such a model, although more complicated, is to our knowledge the only one describing human myometrium sampled from women delivered preterm and provides additional support for other *in vitro *studies.

*In vitro *studies are usually preceding clinical application of drug candidates. As ethical specificity practically excludes testing on pregnant volunteers, there is a hectic need for optimal *in vivo *model. Consideration should, therefore, be given to the uterus of non-pregnant women – where, aparting from the lack of significant rise in oxytocin receptors as seen in late pregnancy [15], most of the regulatory mechanisms remains unaltered (i.e. reaction to vasopressin or prostaglandins). Measurements of intrauterine pressure taken in two distant parts of the non pregnant uterus (isthmus and fundus), thanks to a novel analysis of signals, delivered new data on significant differences in spatial characteristics of contractions [16-18]. Moreover, a model of contractility of non-pregnant uterus may be used for the assessment of the influence of novel drugs reducing uterine contractions. In one of the latest publications, we explored the influence of short time, low dose tamoxifen treatment on parameters of uterine contractile activity of dysmenorrhoeic women [19], showing potentially significant direct influence on parameters of contractile activity; importantly, these would not be detected by conventional methods of signal analysis. Even though tamoxifen treatment may not find its place in obstetrics, the study does illustrate that similar methodology may be applied in the assessments of other drug candidates, such as novel oxytocin antagonists or calcium channel blockers. Multifocal assessments of uterine contractile activity in pregnant women may be performed by the measurements of electric [20] or magnetic [21] activity of the uterus, the novel approach of assessment of uterine magnetic resonance was recently utilized for highly advanced mathematical construction of the virtual atlas of functional biology of human uterus [22]. Special analysis of data may be helpful in establishing interrelations between various parameters of contractions such as synchronization or propagation and clinical outcome.

## Novel analysis of signals in studies on preterm labor

Our advanced methods of signal processing and analysis reported here give added support to the potential benefit of new preclinical and clinical models of preterm labor. Such an attitude could provide new insights into "well known" phenomena such as uterine contractile activity. Classical modalities of signal analysis, such as time domain and frequency domain-based analysis are frequently insufficient in providing significant biologic data on uterine contractile activity. In the classical, so called "time domain" analysis, studied parameters including area under curve recording (AUC), maximal amplitude of contractions and various statistical quantities (mean, standard deviation, median, skewness). Although easily computed even for short time recordings [23], these are not sensitive enough to characterize discrete, but potentially important changes in contractile activity. Also, in the frequency domain, the Fourier transform gives no information on the localization of the individual frequency components [24,25], as it provides a globalised integrated from over the whole signal.

Contrary to the Fourier decomposition, the continuous and discrete wavelet transforms allow extracting local and global variations of the recorded contractions. Graphical presentation of such data (scalograms) shows the distribution of energy in the time-frequency plane. For normal contractions, scalograms of the fundal and cervical signals are very similar. In the case of dysmenorrheic contractions, patterns are quite different [25]. Studies on pregnant women using non-invasive approaches are currently in progress.

In the case of bivariate sets of signals (i.e. recorded in two distant locations of the uterus), which contain two simultaneously recorded time series, it appears clinically important to understand how the variables interact. Cross-correlation function can be employed to analyze the association between two time series [18,19,26]. This function is sensitive to the phase differences and can be used to identify time delays (time shifts) between two signals.

**Figure 1:**
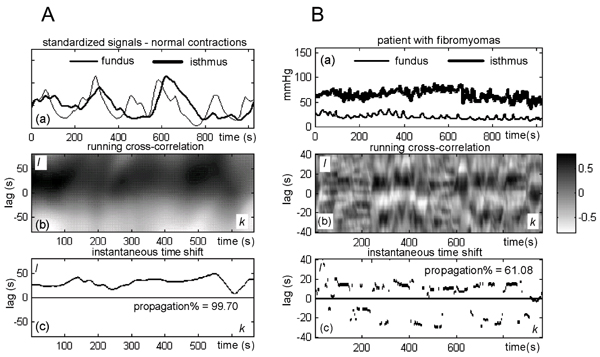
**Graphical presentation of synchronization of contractions of normal (panel A) and fibromyomatous (panel B) uterus**. **Panel A **shows the synchronization of the recording for the normal uterine activity. **(a) **Uterine contractions (standardized signals); **(b) **Running cross-correlation functions (values of the cross-correlation coefficients correspond to the gray levels); **(c) **Instantaneous time shift shows the maximal values of the cross-correlation coefficients for fixed time. **Panel B **illustrates the synchronization for the uterine activity in patients with fibromyomas; **(a) **Uterine contractions; **(b) **Running cross-correlation functions; **(c) **Instantaneous time shift. In **panel A**, the maximal values of the cross-correlation coefficients are mainly located in the area of the positive time lags. In this area there are 99.70 per cent points of the instantaneous time shift curve. So, the propagation% parameter equals 99.70. In this case, the fundal time series mostly runs ahead of the cervical one. In **panel B**, the maximal values of the cross-correlation coefficients are located in the areas of the positive and negative time lags. In the area of the positive time lags are located 61.08 per cent points of the instantaneous time shift curve. In this case we have disturbed synchronization.

Time shifts may be an important indicator of propagation of uterine contractions, which could be altered in women in preterm labor. Figure 1 shows the running cross-correlation and the instantaneous time shifts for normal uterine activity. Standardized sets of signals are obtained after subtraction of the mean from each sample of the signal. The values of the cross-correlation coefficients correspond to the gray levels, as it is shown on the scale bar, which is located on the right of the running map (Figure 1, panel B (b)). Signals of the patient with fibromyomas have disturbed synchronization.

Fractal analysis serves another example for advanced data analysis to be utilized in the studies on uterine contractile activity. Fractals, a relatively new analytical concept of the last few decades, have been successfully applied in many areas of science and technology. We performed a comparative study of ten methods of fractal analysis of signals of intrauterine pressure. We found significant differences between uterine contractions in healthy volunteers and women with primary dysmenorrhoea [17]. There was a correlation between the adjacent elements of the investigated signals. Consequently, the values of fractal dimension can be objective measures for classifications of uterine contraction signals and may be used in studies on preterm labor.

Nonlinear dynamics is one more example of advanced data analysis that could be implemented for uterine contractility signals. In recent years the physiological signals obtained from the complex systems like the brain or the heart have been investigated for possible deterministic chaotic behavior. The human uterus is undoubtedly a complex system. Smooth muscles comprising the myometrium interact in a complex manner. We used the techniques of surrogate data analysis to testing for nonlinearity in the uterine contraction signals. The results showed that the spontaneous uterine contractions are considered to contain nonlinear features [16] which indicated that nonlinear dynamics may increase the accessibility of data for the assessment of biology of uterus, and consequently, the nature of preterm labor.

## Conclusion

After decades of continuous research, especially as no breakthrough in the interest of pharmaceutical industry can be seen, only well-planned and coordinated international programs may provide additional data of different aspects of preterm labor. Possible directions for future research may be related to development of new biophysical methods of evaluation of uterine activity (allowing new pharmacologic studies) together with implementation of novel methodology of analysis of signals and diagnosis of molecular genetic malfunctions.

## Competing interests

The authors declare that they have no competing interests.

## Authors' contributions

All the authors (PP, EO, PL and TL) have made substantive and equal intellectual contributions to a published study and have been involved in drafting the manuscript or revising it critically for important intellectual content.
